# Key Challenges and Opportunities Associated with the Use of In Vitro Models to Detect Human DILI: Integrated Risk Assessment and Mitigation Plans

**DOI:** 10.1155/2016/9737920

**Published:** 2016-09-05

**Authors:** Franck A. Atienzar, Eric A. Blomme, Minjun Chen, Philip Hewitt, J. Gerry Kenna, Gilles Labbe, Frederic Moulin, Francois Pognan, Adrian B. Roth, Laura Suter-Dick, Okechukwu Ukairo, Richard J. Weaver, Yvonne Will, Donna M. Dambach

**Affiliations:** ^1^UCB BioPharma SPRL, Chemin du Foriest, R9 Building, 1420 Braine-l'Alleud, Belgium; ^2^AbbVie, 1 North Waukegan Road, North Chicago, IL 60064, USA; ^3^Division of Bioinformatics and Biostatistics, National Center for Toxicological Research, US Food and Drug Administration (FDA), Jefferson, AR 72079, USA; ^4^Merck KGaA, Frankfurter Strasse 250, 64293 Darmstadt, Germany; ^5^Drug Safety Consultant, Macclesfield, Cheshire SK11, UK; ^6^Sanofi, Bâtiment C. Bernard, 13 Quai Jules Guesdes, Zone B, BP14, 94403 Vitry-sur-Seine Cedex, France; ^7^U.S. Food and Drug Administration, Center for Drug Evaluation and Research, Silver Spring, MD 20993, USA; ^8^Novartis Pharma AG, Klybeckstrasse 141, 4057 Basel, Switzerland; ^9^Hoffmann La-Roche Inc., 4000 Basel, Switzerland; ^10^School of Life Sciences, University of Applied Sciences Northwestern Switzerland, Gründenstrasse 40, 4132 Muttenz, Switzerland; ^11^Ipsen Biosciences Inc., 650 E Kendall Street, Cambridge, MA 02142, USA; ^12^Institut de Recherches Internationales Servier (IRIS), 50 rue Carnot, 92284 Suresnes Cedex, France; ^13^Pfizer R&D, Drug Safety Research and Development, Eastern Point Road, Groton, CT 06340, USA; ^14^Genentech, 1 DNA Way, South San Francisco, CA 94080, USA

## Abstract

Drug-induced liver injury (DILI) is a major cause of late-stage clinical drug attrition, market withdrawal, black-box warnings, and acute liver failure. Consequently, it has been an area of focus for toxicologists and clinicians for several decades. In spite of considerable efforts, limited improvements in DILI prediction have been made and efforts to improve existing preclinical models or develop new test systems remain a high priority. While prediction of intrinsic DILI has improved, identifying compounds with a risk for idiosyncratic DILI (iDILI) remains extremely challenging because of the lack of a clear mechanistic understanding and the multifactorial pathogenesis of idiosyncratic drug reactions. Well-defined clinical diagnostic criteria and risk factors are also missing. This paper summarizes key data interpretation challenges, practical considerations, model limitations, and the need for an integrated risk assessment. As demonstrated through selected initiatives to address other types of toxicities, opportunities exist however for improvement, especially through better concerted efforts at harmonization of current, emerging and novel in vitro systems or through the establishment of strategies for implementation of preclinical DILI models across the pharmaceutical industry. Perspectives on the incorporation of newer technologies and the value of precompetitive consortia to identify useful practices are also discussed.

## 1. Introduction

Drug-induced liver injury (DILI) continues to be a major cause of clinical drug attrition. As such, identification of preclinical models to improve mitigation of this adverse event has continued to be a key focus area among pharmaceutical safety scientists [1–3]. DILI is the major cause of acute liver failure, accounting for ~14% of acute liver failure cases (excluding acetaminophen) with a mortality rate of up to 10% [4–6]. Hepatic injury is a potential clinical adverse finding for orally administered, small-molecule pharmaceuticals due to the anatomical location of the liver, which predisposes it to high transient drug concentrations (“first-pass effect”), and due to its role in xenobiotic metabolism and elimination. Therefore, continued efforts to improve preclinical models in terms of prediction and to better understand the translational implications of risk factors identified preclinically remain a major priority and challenge.

Intrinsic DILI typically occurs at a high incidence, will usually manifest in both animals and humans when a drug is taken at sufficiently high doses, and has an acute onset. As such, current preclinical models commonly detect drugs causing intrinsic DILI. The outcome is that severely hepatotoxic drugs are discontinued during discovery or early development phases, and those advanced to the clinic have safety margins that are considered acceptable for the intended indication. In contrast, idiosyncratic DILI (iDILI) occurs with less frequency ranging from an incidence of 1 in 100 patients (e.g., chlorpromazine) to the more typical incidence of 1 in 10,000 patients (e.g., flucloxacillin). Furthermore, iDILI does not follow a predictable dose-response relationship, is not related to the intended pharmacology, and often has an unpredictable or latent onset often occurring after weeks or months of dosing. Finally, iDILI is not reliably detected in preclinical models and thus is the major cause of late-stage clinical trial failures and marketed drug withdrawals [7, 8].

**Figure 1:**
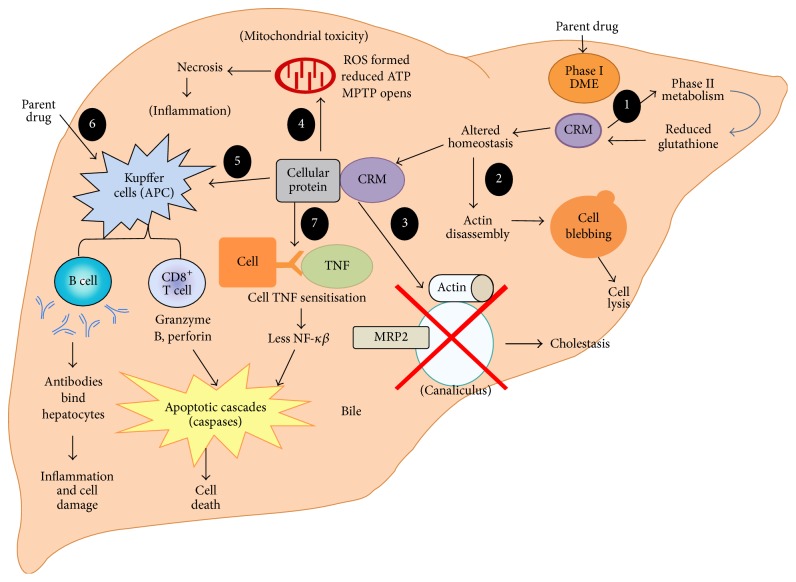
Overview of mechanisms of DILI. Figure extracted from Godoy et al. [21]. (1) Detoxification: conjugation with glutathione. (2) Altered calcium homeostasis. (3) Reactive metabolites may bind to transport pumps or actin around the bile canaliculi preventing bile export. (4) Reactive metabolites binding to mitochondrial proteins may reduce ATP formation, produce ROS, and open the MPTP causing apoptosis. (5) Immune stimulation via the hapten or prohapten mechanisms leading to either humoral (B cell) or cell-mediated (T cell) reactions. (6) Immune activation (PI mechanism with parent drug). (7) TNF receptor sensitivity may be heightened increasing responsiveness to TNF, leading to apoptosis. For more details, please refer to Godoy et al. [21]. Figure reproduced with permission.

The pathogenesis of iDILI is not understood; however, a leading hypothesis posits that there is an initial, intrinsic insult caused by the drug followed by an adaptive response [9, 10]. According to this hypothesis, the initial insult is minimal and subclinical or transient in the majority of the population, whereas the insult is amplified or the adaptive response is inappropriate leading to severe toxicity in susceptible individuals [8, 11]. In particular, evidence suggests that intrinsic, drug-specific drivers of toxicity include drug exposure levels and inherent chemical properties, whereas factors that enhance susceptibility are specific to an individual and include a combination of physiological, environmental, and genetic risk factors [12]. The clinical manifestation of iDILI is related to some threshold concurrence of these independent factors [13, 14]. The physicochemical and structural features of a drug can cause toxicity through metabolic bioactivation and covalent binding to cellular components leading to cellular dysfunction or an immune response and/or by inhibition or alteration of cellular functions. The cellular processes that are commonly affected with DILI include mitochondrial functional impairment and initiation of apoptosis; alteration of protein function (e.g., enzymes or transporters); alterations in redox status; and activation of an immune or inflammatory response as illustrated in Figure 1 [9, 10, 15–21]. Susceptibility factors in individuals influence the adaptive responses to drug injury. The most common factors that have been identified include age, gender, nutritional status, comorbidities, drug-drug interactions, and genetic/epigenetic variability.

Specifically, several key risk factors have been identified through clinical epidemiological studies of drugs causing DILI as follows:

1. Metabolism: drugs with extensive hepatic metabolism (≥50%) have a greater association with elevated alanine transferase (ALT) values (>3 × upper limit of normal), hepatic failure, and mortality [22].
2. Dose: more than 75% of drugs that cause DILI are used at a daily dose ≥50 mg [22–25].
3. Biliary elimination: drugs eliminated via biliary clearance have a higher incidence of jaundice [22].
4. Gender and age:
  - cholestatic DILI occurs with a slight predominance of older age males;
  - hepatocellular (necrotic) DILI occurs predominantly in younger age females;
  - autoimmune-type DILI is reported to occur exclusively in women [24, 25].
5. Hepatocellular DILI: hepatocellular DILI is the most common form to progress to liver failure [25].
6. Genetic polymorphisms: genetic variants of metabolic pathways, inflammatory/immunological pathways, and mitochondrial functions have been reported; often multiple polymorphisms are present [25].
7. Comorbid liver disease: diabetes and viral infections have been associated with enhanced susceptibility [26].

Given the pathogenic complexity of DILI, it is implicit that no single preclinical endpoint or model can predict its occurrence. Instead, preclinical hazard identification and risk assessment will require the integrated evaluation of several endpoints. However, the clinical risk factors and drivers of toxicity are still largely unknown, which hampers the development of predictive preclinical models. This is due, in part, to the fact that there is no definitive clinical diagnostic tool or set of risk factors which defines or predicts iDILI [12], and although various clinical causality-scoring criteria have been established, they are inconsistently used and cannot prospectively predict development of iDILI [26]. In addition, there is a poor correlation between results of animal studies, including rodent and nonrodent species, with the actual clinical outcome for DILI being documented [27]. Furthermore, animal studies are not statistically powered for the detection of low incidence events and are conducted using normal, young and healthy animals that are of similar age. As such, these in vivo studies may not cover many of the susceptibility factors that have been associated with the development of iDILI.

In vitro models can potentially address some specific limitations of in vivo models by leveraging, for example, cells with specific genetic polymorphisms or cells from patients with preexisting liver diseases or known DILI susceptibility. However, most of the currently used in vitro liver systems (e.g., monolayers of hepatic cell lines or primary hepatocytes) do not adequately reproduce the complex physiology of the liver and cannot reflect some mechanistic aspects or environmental conditions under which clinical DILI might occur. Furthermore, there has been no concerted effort at harmonization of current, emerging, and novel in vitro systems or the strategies for their implementation across the pharmaceutical industry. As a result, the knowledge of the utility and performance of the current in vitro systems is limited.

The recent breakthroughs in generating induced pluripotent stem cells (iPSCs) from selected populations may provide the variety of differentiated human liver cell types that will be needed for development of more physiologically relevant test systems [28], despite the current technical hurdles that affect reprogramming and differentiation of iPSC into mature phenotypes. Additionally, complex in vitro systems (e.g., 3D cultures containing hepatocytes and nonparenchymal cells) enable longer incubation times that may better reflect liver physiology [29, 30]. However, these systems still are not evaluated with respect to reproducing the intra- and extrahepatic variety of events (known and unknown) that ultimately lead to iDILI in patients. Future trends are moving toward the use of multiorgan cell culture systems to enhance the physiological relevance of cell cultures [31], as well as cell cultures obtained from diseased patients that may be susceptible to a specific compound. However, these advanced cell culture systems are still at an investigational stage.

The progression of preclinical assessment of DILI, in particular iDILI, will require continued mechanistic investigations both preclinically and clinically. This paper provides an overview of the key challenges for currently available in vitro preclinical models to assess DILI risk, practical considerations for improving the use of these models, and a forward-looking perspective of the opportunities for the use of in vitro models including collaborative efforts to evaluate and standardize the use of these models.

## 2. Promises and Drawbacks of In Vitro Assays

### 2.1. Introduction

A variety of cellular models have been described and illustrative examples are summarized in Table 1. These include relatively simple cell systems that use liver-derived cell lines which express metabolic activity (HepaRG) or have limited (HepG2) or no (THLE) metabolic capacity, transfected cell lines which express physiologically relevant human cytochrome P450 (CYP450) activities, primary hepatocytes cultured in a static monolayer configuration, hepatocytes cocultured with nonparenchymal liver cells or other accessory cells, human liver microtissues that contain multiple cell types in physiologically relevant 3D configuration, and 3D multicellular culture formats exposed to shear stress using microfluidic devices. All of the cellular models can be used as high volume routine assays, apart from human hepatocyte covalent binding studies (which require availability of radiolabeled drugs) and the 3D microfluidic human liver models [32]. Among the cell lines, HepaRG cells represent a highly differentiated model of liver metabolism and transport function for the study of many intracellular events associated with drug toxicity [33, 34].

Based on our current understanding of DILI mechanisms, it is reasonable to assume that an optimal discovery test cascade could require routine high volume use of several assays in parallel, thereby concurrently investigating key mechanisms that may cause DILI. Use of multiple assays that explore individual mechanisms is resource intensive but is essential to develop the required scientific understanding and to enable project teams to explore and understand potential structure-toxicity relationships that can aid rational design of nonhepatotoxic drugs. Such assays are also valuable for exploring and understanding mechanisms by which drug candidates cause liver injury in humans or animals and potentially to enable selection of alternative compounds that do not exhibit such liabilities.

### 2.2. From Patients to In Vitro Early Screening: The Promise of hiPSCs

The generation of functional hepatocytes from human induced pluripotent stem cells (hiPSCs) continues to pose a major challenge. Although iPSC-derived hepatocytes have been generated, these remain neither fully characterized nor validated, and currently these cells cannot be produced on a large scale. Nevertheless, cardiomyocytes derived from human cells are currently in use and provide valuable insight into the usefulness to the pharmaceutical industry of differentiated cells derived from hiPSCs.

The classical preclinical methods for detecting cardiotoxicity have relied on genetically modified cell lines, which do not accurately simulate human cardiomyocytes. Recent technological advancements permit the generation of hiPSCs from the skin, which can then be used to produce patient-specific cardiomyocytes (CMs) under in vitro conditions. This means that each hiPSC generated from a patient's fibroblasts carries the relevant genetic information from that individual, thereby providing a huge opportunity to better understand many human disorders through “disease in a dish” modelling. For example, hiPSCs have been used to recapitulate disease phenotypes of genetic cardiac diseases such as long QT syndrome (LQT [35]), familial hypertrophic cardiomyopathy (HCM [36]), and familial dilated cardiomyopathy (DCM [37]). Patients suffering from LQT, HCM, and DCM syndromes are particularly sensitive to cardiotropic drugs and are vulnerable to fatal arrhythmias [38]. Recently, a library of hiPSC-CMs derived from patients with LQT, HCM, and DCM was characterized and screened against a panel of drugs known to affect cardiac ion channels [39]. Liang and collaborators [39] recapitulated drug-induced cardiotoxicity profiles for healthy subjects and LQT, HCM, and DCM patients at the single cell level for the first time. The data obtained revealed that healthy and diseased individuals display different susceptibilities to cardiotoxic drugs [39]. In other words, cohorts of disease-specific hiPSC-CMs have produced distinct pathological phenotypes associated with clinical presentations of LQT, HCM, and DCM. Finally, Liang et al. [39] revealed that hiPSC-CMs could detect drug-induced cardiac toxicity more accurately than the classical preclinical assays mandated by regulatory authorities.

These investigations using iPSCs clearly illustrate the ability to use these models for lead optimization and exemplify the concept of personalized medicine using in vitro assays, which enable assessment of the genetic susceptibilities of distinct individuals to better predict clinical outcomes. This aspect is especially valuable, because the majority of cardiotoxic drugs have a low incidence of harmful effects for the general population (similar to DILI) and are often toxic to specific patient populations with determined genetic traits [39]. Taken together, these findings strongly support the use of hiPSC-CMs to better select and develop promising compounds devoid of cardiotoxic effects.

### 2.3. Generation of Human In Vitro Data to Predict Clinical Data: A Case Study with Fialuridine

Second generation nucleoside analogues, such as fialuridine (FIAU), have been used as potential drugs to treat hepatitis B. Preclinical studies in mouse, rat, dog, and monkey showed no sign of DILI at doses up to 1000-fold the human therapeutic dose [40, 41]. In a clinical trial, fifteen patients with chronic hepatitis B received FIAU at a dose of either 0.10 or 0.25 mg kg/day for 24 weeks and were monitored every 1 to 2 weeks by means of physical examination, blood tests, and testing for hepatitis B virus markers [42]. Unfortunately, seven patients developed severe hepatotoxicity, with progressive lactic acidosis, worsening jaundice, and deteriorating hepatic synthetic function [42]. Five patients died and two survived after liver transplantation. These toxic effects were probably caused by mitochondrial damage and were not predicted by animal studies [42]. In vitro investigations using hepatocytes in a micropatterned coculture model (Hepregen Corporation) revealed that FIAU was significantly more toxic to human hepatocytes (IC_50_: ~5 *μ*M) as compared to rat hepatocytes (IC_50_ > 100 *µ*M), while its diastereoisomer was not toxic (IC_50_ > 100 *µ*M) in either species [43]. These data illustrate the added value of using human relevant models as a part of the selection of drug candidates because in vivo preclinical studies do not always predict clinical outcome. A large multinational pharmaceutical company survey, which evaluated animal toxicity data and human adverse effects observed in clinical trials of 150 candidate drugs, revealed a true positive human toxicity concordance rate of 71% for rodent and nonrodent species [44]. Toxicity studies in nonrodents alone were predictive of 63% of the 221 human toxicities that were observed, while studies in rodents alone were predictive of 43%. Furthermore, DILI and hypersensitivity/cutaneous reactions in humans were the most difficult target organs to predict based on animal studies [44]. Therefore, there is a substantial opportunity for data provided by well-validated in vitro models to improve human DILI prediction.

### 2.4. Drawbacks and Limitations of In Vitro Assays

Useful in vitro assays should focus on detection of known mechanistic risk factors for DILI in humans. An important use of these assays is to flag and enable deselection of compounds exhibiting a high human DILI propensity, thereby aiding the selection of drug candidates with low propensity to cause DILI. It is now generally accepted that interpretation of data provided by in vitro assays requires knowledge of in vitro drug potency (typically expressed as EC_50_ or IC_50_) and can be improved when human drug exposure is available [45–47]. Typically, steady state drug concentrations in plasma (*C*_ss_) or maximum plasma drug concentrations (*C*_max_) are used. Ideally, obtaining in vitro intracellular drug concentrations would be useful when analyzing the data; however, this is usually not known. Knowledge of in vitro hepatocyte concentrations would add important information for understanding exposure-effect relationships, so this limitation is an important consideration.

While the modest DILI sensitivity of individual assays is not surprising since liver injury can occur by different mechanisms, it highlights the limitations of these in vitro models. Development of DILI in patients is a complex consequence of multiple contributory biological processes, all of which are not reproduced by the currently available in vitro methods. Notable omissions include limited or no metabolic capacity, which may result in underestimating toxic effects of metabolites or the potential for detoxification, limited bile formation and excretion, and no adaptive immune responses. Consequently, several groups have explored whether improved sensitivity of DILI prediction can be obtained by combining data provided by several assays, each of which address differing mechanisms. This approach has yielded very encouraging results (e.g., [48]), as have approaches that combine in vitro assay data with physicochemical properties of drugs and/or in vivo plasma exposure data (e.g., [47, 49]).

The cell types utilized in assays are also an important consideration. Primary cells are considered to be the more relevant cell type because they more closely mimic the normal hepatocyte in vivo with regard to expression patterns and functions. Primary cells usually are less abnormal in their overall biology compared to transformed cells lines, which are derived from tumors and continue proliferating even after reaching confluency in monolayers [50]. However, because primary cells do not divide, their supply can be limited and there is a high degree of donor variability with regard to gene expression and function caused by underlying diseases, as well as life style (alcohol abuse, smoking, and chronic drug treatments). Many different types of immortalized liver-derived cell lines are readily available and can be used to study hepatotoxicity. Most hepatocyte cell lines are derived from hepatocellular carcinomas and exhibit abnormal karyotypes and expression patterns that change after passaging of the cells. For example, HepG2 cells display a highly abnormal hyperdiploid karyotype with 55 chromosome pairs (http://www.hepg2.com/) and a long list of genetic mutations [51]. Thus, transformed cells are considered to least represent the normal hepatocyte in vivo and this must be considered when utilizing these cells [50]. Liver cell lines can also be generated from primary liver cells, which can be engineered to become immortalized [52].

Traditional static in vitro cell systems use single cell types and so lack interactions between different cell types (e.g., nonparenchymal and immune) and exposure to immune, hormonal, and humoral factors that together alter liver function [53, 54]. The interaction with the immune system often plays a key role in human iDILI [8] and typically does not occur in toxicity studies undertaken in animals or in traditional monoculture in vitro models. In addition, hepatocytes require key interactions with extracellular matrix components for normal function. This is demonstrated by hepatocytes taking on a pseudo-3D shape and forming functional bile canaliculi when cultured in matrix sandwich configuration but not when cultured in a standard monolayer configuration [55, 56]. Other important factors that impact the use of in vitro models include the choice of dose range and duration of treatment of cells with test compounds, often markedly different from those that occur when patients are dosed with drugs [57], and the physiological cell status, specifically with regard to oxygen tension, which can have important consequences on cell behavior [58].

## 3. Lack of Standardization in the DILI In Vitro Field

A current critical hurdle is the lack of standardization of these models, which limits our understanding of how to best utilize them and the need for validation for potential use in regulatory submissions [59]. The following sections address important parameters which need to be standardized, to facilitate comparison across in vitro DILI studies and thus to maximize scientific knowledge and the potential for industry wide acceptance. Finally, in order to be widely used by the industry, the developed assays will need to be of reasonable throughput, reliable, robust, easy to handle, reproducible, sensitive, specific, cost-effective, and easy to interpret (i.e., with a minimal amount of ambiguity in the data generated).

### 3.1. Compound Classification

**Figure 2:**
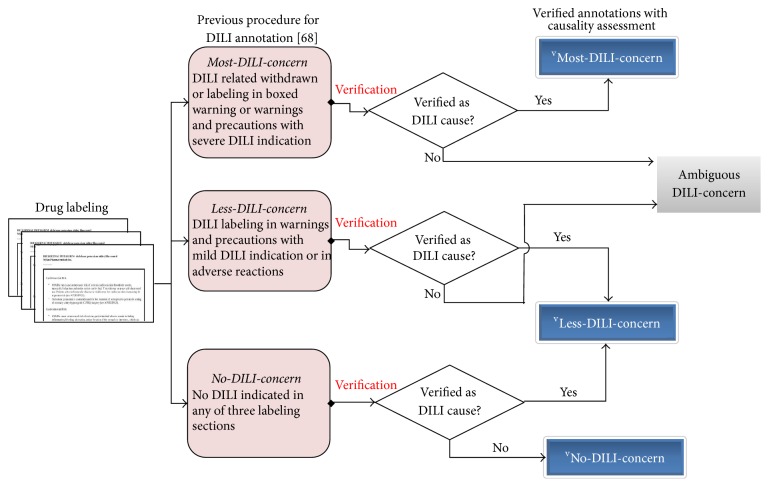
The schema to refine the drug labeling based DILI annotations by weighting the causality evidence. Three verified categories (^v^Most-, ^v^Less-, and ^v^No-DILI-concern) and one “Ambiguous DILI-concern” group were classified in the new schema. For more details, please refer to Chen et al. [65]. Figure reproduced with permission.

The foundation for establishment of an in vitro tool to predict DILI should ideally rely on a well-defined set of compounds, which have been tested in vivo (animal and/or clinical data, depending on what endpoint the in vitro tool is aiming to predict) and where the severity and frequency of observed toxicity are described consistently. For DILI, a key challenge is the need to take account of both intrinsic (acute, short-term) hepatic injury and iDILI. Drugs causing human iDILI are especially difficult to classify because preclinical toxicity data are often not available in the scientific literature and there is only limited knowledge about their clinical adverse effect if available, due to the very low number of patients affected. Furthermore, different investigators may classify the available data in markedly different ways (see Section 5.1 for more details). The following example illustrates the challenge when attempting to classify iDILI. Tacrine was the first centrally acting cholinesterase inhibitor approved for the treatment of Alzheimer's disease, but its use was discontinued in the US in 2013 due to hepatotoxicity concerns. Tacrine has been classified by different investigators as nonhepatotoxic [60], moderately hepatotoxic [61], or highly hepatotoxic [62, 63]. In the Liver Toxicity Knowledge Base (LTKB), it is classified as ^v^Most-DILI-concern with a DILI severity score of 7 [64], because rare cases of liver toxicity associated with jaundice, raised serum bilirubin, pyrexia, hepatitis, and liver failure have been reported in Tacrine exposed patients (LTKB data). This example demonstrates the conflicting information available for compound classification (for more details please refer to Chen et al. [65], Figure 2 and Section 5.1). Classification of the type of hepatotoxicity is also important to consider, especially when investigating mechanisms of action, as there are many different liver pathologies caused by drugs [66] (e.g., liver hypertrophy, bile duct hyperplasia, cholestasis, steatosis, and phospholipidosis). The link between the liver specific pathologies and mechanism of actions is largely unknown now, but its exploration will be important to help better understanding and prediction of hepatotoxicity. Finally, it is important to recognize that the DILI classification of a given drug may evolve with time as new information becomes available.

The current lists of DILI drugs used for the validation of in vitro models contain a mixture of compounds with high and very low incidence for DILI, as well as intrinsic and idiosyncratic toxicants [67]. This mixture of incidence and type of DILI confounds the predictive power of these assays. A more realistic approach for assessing the predictive value of a new assay would be to separate model compound sets based on their incidence of injury [67]. To achieve this, a collaborative effort is required to obtain and share incidence data and to determine cut-offs for inclusion of compounds as positive or negative controls [67]. It is important to use a reliable and recently updated system that allows for classification of drugs. The LTKB was developed with the specific aim of enhancing our understanding of DILI ([65, 68] and Section 5.1). It is recommended that current and future investigators use the LTKB to aid their compound selection and data interpretation wherever possible, thereby enabling improved comparison between different models. It is also proposed that researchers use a well-balanced selection of reference drugs spanning a wide range of targets and chemical structures, in order not to bias the training set. The chemical space in drug development has dramatically evolved over time and many of the new drug entities in industry display properties which are potentially not represented in reference sets of well-characterized classic hepatotoxic drugs developed decades ago. This poses a risk for both under- and overprediction of hepatotoxic potential.

### 3.2. Concentrations and Cut-Off Selection to Evaluate the Predictivity of In Vitro Models

The translation of exposure-effect relationships from in vitro to in vivo is a major challenge for drug testing. For creation of a reference set with well-known drugs, clinical exposure data should be incorporated to mimic liver drug load as closely as possible. As outlined below, some published approaches make use of such concentration estimates. What needs to be taken into account is the fact that, at the stage of development, where a DILI assay typically would be applied, human exposure data is not available, and in most cases animal exposure data are not available. In vitro pharmacology data and ADME parameters can be used to estimate human exposure, with the caveat that these estimates have a significant degree of uncertainty, which, in turn, limits the conclusions that can be drawn about the translatability of a toxicity signal at a given concentration.

Scientists usually assess assay performance in terms of sensitivity and specificity. The sensitivity (true positive rate) is defined as the ability of a test system to predict the positive outcome under evaluation (i.e., hepatotoxicity). The specificity (true negative rate) represents the ability of a test system to predict the negative outcome under evaluation (i.e., nonhepatotoxicity). It is clear that such parameters depend greatly on the concentrations and cut-offs used in the experiments. Some studies have used fixed concentrations to study drugs in the ranges 0.1–100 *µ*M [69], 100 *µ*M [61], 1–500 *µ*M [63], and 1–1000 *µ*M [70] and/or multiples of plasma *C*_max_ (the therapeutically active average plasma maximum concentration value upon single-dose administration at commonly recommended therapeutic doses): 30-fold [61], 1–100-fold [71], 12.5–100-fold [62], and 100-fold [60]. In addition, different concentration criteria have been used to classify drugs as hepatotoxic: 10 *µ*M [69], 100 *µ*M [63], 100 and/or 1000 *µ*M [70], 30-fold [61], or 100-fold *C*_max_ [60, 62, 71]. All together, these data illustrate the diversity in the strategies in terms of concentrations and cut-offs. They may also reflect an attempt to set thresholds that best fit the experimental data to obtain the most favorable predictivity in terms of specificity and sensitivity outcomes. However, these adjusted thresholds may not hold true with a different set of data. Hence, it would be helpful to reach a consensus particularly when reference drugs are used. Xu and collaborators [60] reported that the 100-fold *C*_max_ scaling factor represented a reasonable threshold to differentiate safe versus hepatotoxic drugs. This calculation takes into account different scaling factors: 6 × (for population *C*_max_ variability), 6 × (for higher drug exposure to the liver), and 3 × (for drug-drug or drug-diet interactions) = 108  *C*_max_ which has been approximated to 100 *C*_max_ [60]. For screening activities, in absence of known *C*_max_ values, fixed concentrations therefore should be used. When analyzing and interpreting data obtained for drugs which have been evaluated in the clinic, it is more logical to use multiples of *C*_max_ up to 100-fold as this is scientifically justified. However, one limitation of using a *C*_max_-based testing approach is that it does not take into account potential drug accumulation in the liver or protein binding (see Section 5). Nevertheless, *C*_max_ values can be easily measured and are easily accessible for reference compounds.

### 3.3. Endpoint Selection

Liver injury is certainly challenging to predict because many mechanisms can induce hepatotoxicity (Figure 1) and what finally results in DILI may be the interplay of genetic disposition of the patient age and disease state and a chain of cellular effects triggered by drug treatment leading to multiple events. The types of DILI cellular events can be very diverse (see introduction part for more details). So before using any in vitro models it is important to determine which mechanisms can be detected, particularly when these are used as a part of investigative studies.

**Figure 3:**
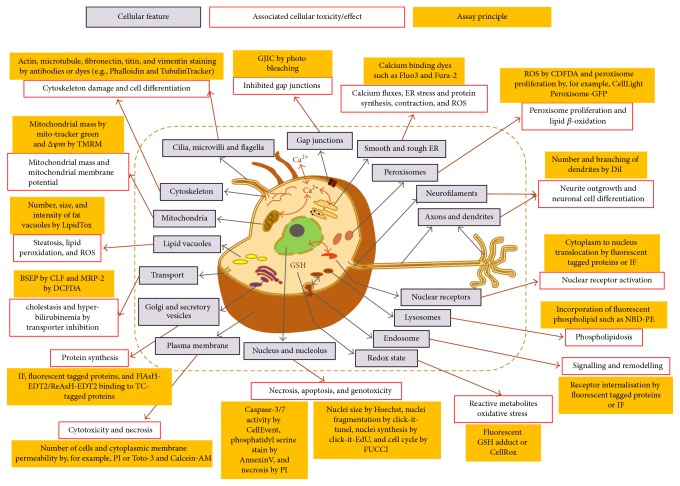
HCI assay examples for assessment of specific cellular functions and toxicity. For more details, please refer to Uteng et al. [81]. Figure reproduced with permission.

Many diverse endpoints have been measured such as ATP [48, 62], LDH [72], 5-carboxyfluorescein diacetate acetoxymethyl ester [73], albumin [74], impedance (label free approach) [62], glutathione [62, 71, 75], reactive oxygen species [75], mitochondrial toxicity [76–78], phospholipidosis [79], transporter inhibition [48], or a mixture of parameters using high content analysis [55, 70, 80, 81] as illustrated in Figure 3. Screening compounds using high content imaging of cells have the advantage of measuring multiple parameters simultaneously. For instance, Persson et al. [80] presented the validation of a novel high content screening assay based on six parameters (nuclei counts, nuclear area, plasma membrane integrity, lysosomal activity, mitochondrial membrane potential, and mitochondrial area). Multiple parameters can also be measured with other approaches. For example, glutathione and ATP levels as well as albumin and urea secretion were measured in micropatterned coculture models [71]. In this study, it was reported that albumin secretion was the most sensitive parameter (10/10), followed by urea secretion and ATP levels (9/10) and GSH levels (7/10). Consequently, nondestructive measurement of albumin and urea in medium could be sufficient for an initial toxicity assessment, whereas parameters such as GSH could be used subsequently for probing specific mechanisms [71]. In another study, Porceddu and collaborators [82] developed a high-throughput screening platform using isolated mouse liver mitochondria and measured multiple mitochondrial endpoints such as inner and outer membrane permeabilization as well as alteration of mitochondrial respiration driven by succinate or malate/glutamate.

It may not be possible to reach a consensus on a list of markers to use to measure hepatotoxicity in vitro. One may also question the relevance of measuring general cytotoxicity markers in comparison to more mechanistic endpoints. Nevertheless, scientists are encouraged to use endpoints that cover as many mechanisms as possible in a logical and hypothesis-driven manner as illustrated by Thompson et al. [48]. Next to technologies allowing parallel measurements in one experiment such as high content imaging, approaches incorporating a battery of assays run in parallel and taking into account exposure aspects have been recently published and show promising performance with respect to DILI prediction [48, 83].

### 3.4. Other Parameters Influencing the Predictivity of DILI In Vitro

*Length of Exposure*. Short-term, high dose, single exposure in vitro studies are often performed but they have missed a number of hepatotoxic drugs in humans. One reason could be that the exposure time is restricted to days while liver injury can occur 1–6 months after initiating therapy [16]. With the emergence of novel in vitro models that can be cultured for weeks, in vitro studies with repeated administrations are now more common [62, 71, 84]. For instance, Khetani et al. [71] reported that more hepatotoxic compounds were detected in coculture models after 9 days of dosing (four repeat drug administrations in total) compared with 5 days of dosing (two repeat drug administrations in total). In another study, the use of label-free technologies allowed longitudinal assessment of cell behavior from attachment to the end of experiment and after compound additions [62]. The next step will likely be to expose in vitro models to low doses of drugs for longer time to better mimic the human situation. Finally, the selection of endpoints, the duration of exposure, and the number of repeat drug administrations chosen for these in vitro models is also a matter of debate. One may consider that, in a screening mode, single administration and 24–72 h exposure may be enough to rank compounds, whereas multiple administrations over long periods may be required for mechanistic studies to better compare to in vivo data.

*Culture Conditions*. The objective here is certainly not to describe all factors that influence the data but simply to remind scientists that a simple change in culture conditions may have a strong impact on the data generated. For instance, the presence of serum may not only decrease drug free concentrations due to protein binding but also enhance the long-term culture of coculture models [71]. In addition, most cell media contain high concentrations of glucose. As a consequence, ATP is mainly generated via glycolysis despite the presence of oxygen and functional mitochondria in cells. Unfortunately, such anaerobically poised cells are resistant to xenobiotics that impair mitochondrial function [85, 86]. To better allow the detection of drug-induced mitochondrial effects, it is important to force cells to rely on mitochondrial oxidative phosphorylation rather than glycolysis by substituting glucose with galactose in the growth media forcing cellular use of glutamate through the Krebs cycle [86]. Another important parameter to consider is the solvent used to solubilize test materials and especially the concentration of solvent in order not to interfere with cell functionality. For example, while DMSO is a commonly used solvent, it is known that above a certain concentration DMSO may have an effect on mitochondria and CYP activities and may modify cellular responses [87], as well as being an antioxidant which may also hinder the effect of reactive oxygen species [88].

*In Vitro Models*. There are a large number of in vitro and ex vivo models available (e.g., 2D, 3D, with or without nonparenchymal cells, static, microfluidic, microtissues, liver slices, and perfused liver), but currently there is no clear consensus on which models most accurately predict human hepatotoxicity. While many systems such as liver slices and isolated perfused livers have been developed to investigate mechanisms of liver toxicity, technical, economical, and reproducibility issues limit their use in drug discovery where reliability and throughput are key factors. All models have strengths and weaknesses and one may argue that a particular model may be better to detect some of the DILI mechanisms, but none of the models address all mechanisms. Furthermore, there is a lack of agreed upon controls in such experiments and descriptions of experimental parameters such as oxygenation (see Section 2.4 for more details) and cellular functionality, including target expression and metabolism (xenobiotic metabolism and energy metabolism), are often not provided; these should be included in the experimental design. Even if the same cells are used across different studies, cells may not remain in the same experimental state, which may partly explain the differences in results reported by different investigators [89].

Three-dimensional and dynamic, microphysiological models are believed to be more physiologically relevant since they more closely reproduce the structure of the organs and physiologic conditions, such as blood flow. The same arguments were used for organ slices and perfused organs, with the difference that ex vivo organ slice cultures come with significant levels of inflammation and tissue necrosis as a consequence of the preparation process. The newer 3D models also have challenges, particularly with regard to the level of oxygen, as hypo/hyperoxygenation may generate toxic artifacts in cells and tissues [90–92]. The prediction of iDILI is even more challenging as it may require individualized in vitro models, as well as a substantial number of tests [93]. Finally, it is important to identify the right model depending on the pathology of interest. For example, prediction of hepatic fibrosis is often based on stellate cell cultures but metabolism-based fibrosis may not be detected in this cell system [94].

When setting up new in vitro assays, the assumption is that the selected in vitro model can recapitulate the mechanism of toxicity leading to DILI, but this may not always be the case. It is now generally accepted that transformed cell lines insufficiently represent hepatocytes. Also, primary hepatocytes cultured for just 24 h in monolayers only partly display the complexity of biological interactions of native liver. Cocultures and 3D cultures of hepatocytes that permit long-term compound exposure as well as inclusion of other liver nonparenchymal cell fractions increase the chances of detecting liver toxicants which typically escape conventional testing systems [21, 29, 30, 95]. The choice of a particular model may be based on short-term versus long-term culture and the ability to study the toxicity of parent compounds or metabolites [96]. Finally, another aspect is how to address genetic variability in the patient populations. This is clearly challenging and may not be addressed to full satisfaction even when using different hepatocyte donors.

The development of in vitro models representing a pathological state would be highly desirable to enable better prediction of DILI in humans. Of interest in this regard is the use of hiPSC-derived hepatocytes from healthy volunteers as well as DILI patients. Such approach could help the scientific community to better predict human DILI and understand the role played by genetic predisposition. In addition, this may open new opportunities to develop assays using patient-derived hiPSC particularly to better understand individual differences in iDILI susceptibility. Nevertheless, some technical challenges need to be resolved particularly regarding the activity of drug-metabolizing enzymes, as well as the generation of mature and fully functional hiPSC-derived hepatocytes [28].

### 3.5. Concluding Remarks

The scientific community can fuel progress by establishing consensus around reference drugs with recommended test concentrations and cut-off criteria for conducting and interpreting in vitro studies. This would enable comparison of the performance of many in vitro models. Such approaches have already been applied in the field of in vitro genotoxicity (refer to Section 6.3 for more details). Furthermore, a clearer guidance is needed for the classification of reference drugs as severely toxic, less severely toxic, or nonhepatotoxic associated ideally with clear mechanisms of actions and specific histopathology lesions.

## 4. In Vitro Model Characterization and Validation: The MIP-DILI Consortium

Many private and public initiatives have invested extensive efforts toward the development of research tools for the early and safe prediction of human DILI. These efforts have provided biomedical tools for use preclinically and methods for detection and monitoring of DILI in the clinic. Among the many research initiatives are those which have contributed to recent developments of novel biomarkers for use in the identification of DILI [97], novel preclinical animal models [98, 99], and preclinical diagnostics, including “omic” technologies [100, 101] for the early deployment and detection of chemical risk assessment during preclinical R&D. Despite much research in the field of human DILI, little, if any, progress has been made toward a thorough understanding of which of the different in vitro systems that are routinely employed in pharmaceutical research and development are more suited for the detection of certain types of hepatocellular injury [29, 102–104]. To address these questions in conjunction with still poorly understood mechanisms of human DILI, a 26-partner consortium was formed under IMI's EU Industry-Academic Partnership Programme on Drug-Induced Liver Injury and Mechanism-Based Integrated Prediction of DILI [105]. The overarching and primary objective of MIP-DILI is to specifically address the a priori need for an improved panel of in vitro assays for the prediction of human DILI risk of drug candidates during the lead optimization and preclinical candidate selection phases of drug discovery.

MIP-DILI broadly comprises four principal work streams: the evaluation of existing and novel in vitro cell models, biomarkers of cell injury, and mechanistic studies complemented by mathematical modelling approaches for the improved understanding of human DILI. The evaluation of in vitro cell models comprises the quantitative pharmacological, toxicological, and physiological phenotypes of primary human hepatocytes and cell lines, HepaRG and HepG2, in routine use by industry. In addition, novel cell models, such as hiPSC-derived hepatocytes in 2D and 3D cell platforms, are being evaluated in parallel with the overall aim of identifying which of these cell models are more appropriate for the detection of certain types of hepatocellular injury. Biomarkers assessed for use as endpoint measurements of hepatocellular injury include those commonly employed by industry, such as cytotoxicity and mitochondrial dysfunction, alongside novel biomarkers indicative of hepatocellular stress, necrosis, and apoptosis [97]. The quantitative evaluation of toxicological readouts for each of these cell models are supported by use of evidence-based selection of drugs (training compounds) known to cause clinical DILI, together with prevailing mechanisms by which these drugs are believed to cause liver injury. Of the mechanisms currently described, training compounds are grouped according to mitochondrial and lysosomal impairment, intrahepatic cholestasis, immune response, and cytotoxicity. These well-described training compounds are further complemented by a larger set of test compounds to validate the selection of cell models and endpoints.

The combined efforts of interlaboratory ring-trials are enabling in-depth evaluation of different test systems [106] and their comparative sensitivity and selectivity for the detection of certain forms of human DILI. These yet vitally important ring-trials are beginning to provide the industry with important comparative bench-marking of the simplest 2D test systems and direct quantifiable measures of gain-of-physiological and pharmacological function. In addition, any improved sensitivity and selectivity for the detection of chemical risk in more complex 3D formats are being assessed. An important contribution toward the efforts of establishing an improved panel of in vitro assays is efforts by the consortium toward understanding intrahepatic and extrahepatic events leading to hepatocellular injury. These activities include the mapping of primary gene signalling pathways, proteomic and transcriptomic studies, and the direct and indirect effects of drugs on hepatobiliary function. These mechanistic studies, coupled to modelling activities, are helping underpin the characterization of cell models and the pathologies associated with drug toxicities.

A major gap in the current panel of preclinical models available to industry is a test system that affords the detection of immune-mediated human DILI, which is believed to be a central tenant of idiosyncratic drug toxicities. Both the innate and adaptive immune systems are believed to play a role in both the initiation and attenuation of iDILI [107, 108]. The complexity of immune-mediated DILI cannot be underestimated with intra- and extrahepatic signalling and genetic diversity bringing with them important challenges to the development of meaningful test systems for drug safety assessment [105].

Ultimately, the MIP-DILI consortium aims to help defining which test systems are more amenable for use in the detection of certain types of drug-induced hepatocellular toxicities and when to use these test systems during the drug discovery process. To provide solid bench-marking and exploit novel test systems to offer an important step-change for the improved risk assessment of new drug entities prior to preclinical regulatory and early clinical research programmes will be a key deliverable for the project. Now in its 5th year (in 2016), the work performed by MIP-DILI continues to make significant contributions toward a better understanding of the in vitro models most likely to improve pharmaceutical research and development and to define a concrete, tiered roadmap for future research in this field. The MIP-DILI consortium is supported by the Innovative Medicines Initiative Grant Agreement number 115336.

## 5. Practical Considerations

### 5.1. Drug Annotation/Classification

A reference list of drugs annotated for DILI risk in humans is required for the development of in vitro predictive models. The performance of the developed predictive models is subject to the quality of DILI annotations. By DILI annotation we refer here to the classification of drugs based on DILI risk observed in human populations treated for various diseases and reflects the frequency, causality, and severity of DILI for each drug [68]. Information on mechanisms of actions and effects at pathological level are also key parameters to take into account but this level of information is often unknown for the majority of the compounds used in the in vitro investigations. Unfortunately, there is no “gold standard” for defining DILI risk and no consensus on drug classification for DILI. Some authors classify a drug as DILI positive or negative according to the availability of DILI case reports retrieved from the literature [63, 109, 110] or FDA's adverse event reporting system (FAERS) [111–113], while others utilize information summarized in the drug compendiums such as Physicians' Desk Reference [114]. Inevitably, inconsistent annotations based on different approaches are reported for some drugs (as already reported in Section 3.1). For example, buspirone is an anxiolytic psychotropic drug that is used to treat generalized anxiety disorder. The compound has been classified as both nonhepatotoxic [60, 61, 70, 71] and mildly hepatotoxic in humans [62, 63]. Additionally, buspirone was classified as a ^v^less-DILI-concern drug in the Liver Toxicity Knowledge Base (LTKB) with DILI information only found in the label section of “Adverse Reactions” with the query “infrequent increases in hepatic aminotransferases were found during premarketing trial” [68]. Thus, the variability in published DILI annotations, each utilizing different schema and data sources, is an impediment for the development of predictive in vitro models.

The classification of DILI negative compounds is even of more concern. Most published approaches that define a drug as DILI negative depend on search results from PubMed or other databases [60, 61, 70, 71, 113]. In some studies [115], drugs were labeled DILI negative if they simply were without searchable results for a specific DILI adverse event (e.g., acute liver failure). However, due to the diverse manifestations of clinical DILI and the severe underreporting of DILI cases [68], these approaches may miss the information necessary to designate a drug as DILI negative. In a recently published survey, 7.9–41.8% of drugs defined as DILI negative in published datasets were verified as the cause of DILI in case reports in which causality had been fully justified [65]. The high percentages of misclassification highlight the importance of selecting appropriate DILI annotation. Chen et al. [68] published a DILI annotation approach based on FDA-approved drug labeling and classified 287 drugs into three categories (i.e., ^v^Most-DILI-concern, ^v^Less-DILI-concern, and ^v^No-DILI-concern). Recently, the authors refined the drug labeling based approach by incorporating causality evidence collected from the literature and further classified 1036 FDA-approved drugs into three verified categories (i.e., ^v^Most-DILI-concern, ^v^Less-DILI-concern, and ^v^No-DILI-concern) and one “Ambiguous DILI-concern” category (Figure 2) [65].

These drug labeling based DILI annotations are recommended for the development of in vitro DILI models. Firstly, although it is not perfect, the FDA-approved drug labeling is the authoritative document in which drug safety information is summarized through the systematic assessment of data from clinical trials, postmarketing surveillance, and literature publications. The comprehensive information contained in drug labels is especially useful for limiting false negative DILI compounds [65]. Secondly, the procedures of the DILI-label based annotation approaches are transparent and reproducible, and the data source (i.e., drug label and causality evidence) can be updated with the advance of DILI knowledge. Thirdly, the annotations and dataset have been extensively applied to develop in silico [64, 116], in vitro [49, 62, 63, 71, 117–120], and in vivo models [121–124] and were also recommended as the standardized list for model validation [95].

### 5.2. Endpoints

Many pharmaceutical companies have implemented or are developing screening paradigms to decrease hepatotoxicity-related attrition. While some companies try to address this aspect during series selection, others put more emphasis during lead optimization and compound selection. Screens for series selection require the following attributes: appropriate throughput, utility in hazard identification, and utility for rank-ordering of compounds. In a recent publication by Aleo et al. [118] a strong correlation was found between DILI in humans and compounds exhibiting mitochondrial toxicity as well as inhibition of the bile salt export pump (BSEP). Compounds exhibiting both liabilities were more likely to be associated with more severe clinical DILI than compounds with only one of these two liabilities. These data suggest that adding mechanistic endpoints could be useful to decrease hepatotoxicity-related attrition.

Triage of compounds using high-throughput cytotoxicity, sometimes followed or complemented by additional mechanistic endpoints, represents a rather common general approach used by pharmaceutical companies. However, approaches across companies are extremely variable, such that it is not possible to identify the optimal approach. It is noteworthy that the use of liver-derived cell (THLE and HepG2) lines and simple cytotoxicity readouts is likely not sufficient to predict DILI accurately and may be more representative of generalized cell health [125]. Only more liver specific mechanistic endpoints can provide specificity toward prediction of hepatotoxicity.

During hit selection and lead optimization, some companies utilize high content technologies for assessment of specific cellular functions and toxicity (Figure 3). Caution must be taken when utilizing this technology for the assessment of organ toxicity and particularly liver toxicity. The combined measurement of multiple mechanistic endpoints (e.g., mitochondrial membrane potential, ROS formation, ER stress, lipid accumulation, and DNA damage) has limitations for the prediction of liver injury. For example, many compounds known to induce cardiac toxicity will be equally positive using this approach [126]. Hence, to increase predictively toward liver injury, true liver specific endpoints should be assessed, such as transporter inhibition (e.g., BSEP or MRP) [55].

Analysis of new chemical entities (NCEs) using either cytotoxicity assessment [46, 127] or single mechanistic endpoints such as mitochondrial toxicity [78, 82, 128, 129], BSEP [19, 118], or high content analysis [60, 61, 81] is a hazard identification approach that requires exposure information for appropriate risk assessment. Hence, this approach is most useful for comparing and rank-ordering compounds, especially when assessing compounds with similar physicochemical properties. Finally, this strategy is predicated on establishing in vitro systems that could mimic the in vivo biology for functional or morphological events but does not take into account the concentrations needed to achieve them in vitro.

### 5.3. Concentrations, Duration of Exposure, and Culture Conditions

In vitro systems can reproduce in vivo observations, but a correlation between in vitro concentrations and in vivo systemic exposure levels (*C*_max_, AUCs, and bound or free fraction) may not exist. Indeed, drug concentration in the portal vein and liver can be much higher than plasma concentrations and human subjects differ in their drug-metabolizing capabilities and hepatic transporter content/function. Therefore, studies that have been aimed at establishing predictivity for human outcome have tested the compounds at concentrations between 30 and 100 times higher than human plasma *C*_max_ [47, 60–62, 130, 131]. No matter the final endpoints, either straight cytotoxicity or more relevant functional parameters, this approach remains very empirical and arbitrary as many factors can offset the relevant concentration range from in vivo to in vitro settings. First, plasma concentrations may be either significantly above or below tissue concentrations [132]. The drug volume of distribution can indicate tissue accumulation, but assessment of individual tissue exposure to drugs and eventually to their metabolites is rarely performed. In addition, in tissues like liver, drug concentrations can also be vastly different between the various parts of the liver (e.g., centrilobular versus periportal) [133] or the cell types (e.g., hepatocytes versus nonparenchymal cells). This is particularly true for compounds inducing phospholipidosis where cationic amphiphilic drugs with similarity to bile acids [134] can accumulate up to mM range in cells like cholangiocytes while hepatocytes could remain mostly in the nM to *µ*M range. Furthermore, *C*_max_ values are not always the relevant concentrations to be considered when the overall AUC might drive the observed toxicity [135]. In that case, the duration of treatment in vitro should be more relevant than the actual compound concentration, or, more accurately, the combination of both. Increasing the length of treatment in vitro by a few days may not be an issue with cell lines that divide and survive on plastic but is more challenging with primary cells. Finally, a major drawback of a *C*_max_ or AUC-based concentration methodology for “predictive” in vitro screening approaches is that clinical exposures are rarely well estimated at this stage of compound optimization and selection.

The question of the significance of free- versus bond-fraction of the compound is of great importance. In vivo, the drug will have a relatively constant protein binding rate and, most likely, only the free fraction will be the active part for both the pharmacology and toxicology aspects [135]. It is therefore logical to consider protein binding when setting an in vitro dosing range. However, there is great uncertainty of how in vivo free fraction levels translate in vitro. The use of a serum-free culture medium and very well-controlled conditions should be preferred for the following reasons. Firstly, since compounds bind to albumin and other serum proteins, the use of a serum-free medium removes the need to correct for protein binding. However, overall protein concentrations used in vitro are different from the in vivo situation, the binding rates of compounds to fetal calf serum albumin may be quite different from those to human adult albumin [136], and protein binding differs among compounds, especially from different chemical series, limiting compound differentiation. Secondly, compounds can bind the plastic of culture vessels (i.e., dish and tubing of fluidics systems) [57, 137] and this can result in a vast difference between the estimated concentration and the actual medium concentration. Thirdly, as the field moves toward the use of more complex cell culture environments with extracellular matrices [57] (e.g., 3D architectures, cocultures, and fluidics stations), control of compound concentration becomes even more important. Finally, all the above considerations are relevant for chemicals passively diffusing in and out of the cells. For actively transported compounds, the biology of the cells in vitro adds yet another layer of complexity and may skew their exposure, either up or down [132]. Primary hepatocytes typically have lower export transporter and CYP450 function compared to the liver, which may result in overexposure to test articles with some important variations with time [138]. In contrast, many cell lines are transformed tumor-derived cells that may overexpress export pumps such as MDR1 and others, resulting in lower exposures to compounds. Such confounding factors that are only very rarely checked by investigators, as they add a fair burden on the speed and cost of experiments, can lead to erroneous conclusions about the respective cytotoxicity potency of chemicals.

## 6. Examples from Other Disciplines That Could Help to Better Guide Investigations in the In Vitro DILI Domain

### 6.1. The Comprehensive In Vitro Proarrhythmia Assay (CIPA) Initiative

In the early 1990s, six drugs approved by FDA induced cardiac arrhythmia in humans. Consequently, the drugs were withdrawn from the market and international regulatory authorities (US, EU, and Japan) released three guidance documents: two nonclinical (ICH S7A and S7B) and one clinical (ICH E14). Since the implementation of these guidelines, no drugs have been withdrawn from the market due to cardiotoxic events. Nevertheless, these specialized clinical studies add time to development and are very costly [139, 140]. In addition, it is believed that such guidelines prevented some potentially efficacious drugs to reach the market because of false positive signals. In 2013, a workshop was organized to change the current applied cardiac safety arrhythmia guidance paradigm [141]. The proposed paradigm would shift the emphasis from the present approach that strongly relies on QTc prolongation and would obviate the need for the clinical Thorough QT study during later drug development. The Comprehensive In Vitro Proarrhythmia Assay (CIPA), an integrated nonclinical in vitro/in silico paradigm, was initiated toward these aims [142]. CIPA consists of three components aiming to (1) test the effect of compounds on different cardiac ion channels, (2) develop in silico models on cardiac action potential by integrating the ion channel dataset, and (3) measure action potential in human stem cell-derived ventricular cardiomyocytes.

*What Can Be Learned from the Current CIPA Initiative to Help the In Vitro DILI Field?* International consensus on assay protocols, method standardization, and validation will need to be implemented in a new guideline [142]. For instance, a first objective of the three CIPA core assays is to rapidly achieve ICH regional (Europe, Japan, and USA) consensus on best practice protocols (e.g., stimulation rate, holding potential, and specific ion concentration in the pipette solution). The topics of discussions also include the use of either hiPSCs or human embryonic stem cells, cell purity (e.g., proportion of atrial, ventricular, and nodal cells), maturity of the ventricular cells, known limitations of the cells, electrophysiological characteristics of the cells, endpoints (i.e., technology to use), and risk predictability [142]. Although there are currently a high number of in vitro models to predict DILI (e.g., 2D, 3D, stem cells, and liver on a chip) compared to the CIPA initiative, some of the concerns (e.g., cell characterization, optimized protocols, advantages, and limitations of cellular models) highlighted in the CIPA initiative can be directly translated to the in vitro DILI field.

### 6.2. Past Microarray Initiatives

DNA microarrays emerged in the public scientific domain in the early 1990s. Such technology enabled study of the expression of thousands of genes in a single experiment. Initially, no major concerns were described with regard to data analysis, validation, and comparison but the situation changed in the early 2000s. Indeed, the scientific community started to question the influence of many parameters to interpret microarray data, as well as the lack of comparison among different studies [143].

Brazma et al. [144] presented a proposal, the Minimum Information About a Microarray Experiment (MIAME), which described the minimum information required to ensure that microarray data could be easily interpreted and that results derived from its analysis could be independently verified. MIAME has not only facilitated data sharing but also guided software development [145]. In 1999, the Microarray Gene Data Expression Society (MGED) was founded with a basic aim to standardize the field [146]. In addition, MGED asked for the depositary of primary experimental data into a permanent public database. In 2002, the MGED society convinced high impact scientific journals such as Nature, The Lancet, and Cell to require MIAME for publication of microarray results [146].

*What Can Be Learned from the MIAME Initiative to Help the In Vitro DILI Field?* In 2006, a thorough analysis of widespread microarray platforms by a multicenter consortium demonstrated intraplatform consistency across test sites, as well as a high level of interplatform concordance in terms of genes identified as differentially expressed [147]. Since the majority of scientific journals require that raw and normalized microarray data be accessible to the public at the time of publication, a significant number of datasets are publicly available [148]. The technology has been successfully used for disease diagnosis and prognosis, human disease subtype classification, and therapeutic treatment selection.

Overall, the efforts to standardize the microarray field enabled microarray-based gene expression profiling to evolve into a mature, high-throughput, analysis approach that has been extensively applied in biomedical and clinical research for more than 20 years [149]. We believe that the efforts provided in the microarray field could also be used as a relevant example for the in vitro DILI community.

### 6.3. Strategies to Reduce Rate of False Positive in the In Vitro Genotoxicity Field

In vitro genetic toxicology tests are performed for regulatory purposes to predict carcinogenic potential of drugs, chemicals, food additives, and cosmetic ingredients. If a chemical is positive in one of the battery of assays, in vivo genotoxicity studies are often performed to better assess carcinogenic risk for humans. Kirkland et al. [150] evaluated the performance of a battery of three in vitro genotoxicity assays to discriminate rodent carcinogens and noncarcinogens from a large database of over 700 chemicals and found that 93% of rodent carcinogens were detected by the assay battery. Nevertheless, approximately 80% of the 177 noncarcinogenic compounds tested gave a false positive result in at least one in vitro test [150]. The low specificity data highlighted the need for more meaningful in vitro genotoxicity tests or practical interpretation of current positives. In order to address the high rate of false positive results, a 2-day workshop was hosted and sponsored by the European Centre for the Validation of Alternative Methods (ECVAM) in 2006 [150].

*What Can Be Learned from the ECVAM Initiative to Help the In Vitro DILI Field?* The recommendations of the experts were to use cell systems that are p53 and DNA-repair proficient, with defined phase I and phase II metabolic capacities, and to reduce the top concentrations and the maximum level of cytotoxicity to reach [150]. A few years later, Kirkland et al. [151] published recommendations on chemicals that would be appropriate to evaluate the sensitivity and specificity of new/modified mammalian cell genotoxicity tests, in particular, to avoid misleading positive results.

Some of the recommendations expressed in the vitro genotoxicity field could be also applied to the DILI vitro domain. In particular, it would make sense to recommend a list of human hepatotoxicants as well as nonhepatotoxicants to be tested in the different cellular models. A range of concentrations to test per compound would also certainly facilitate the comparison of in vitro DILI studies. In addition, metabolic data on the most relevant cytochrome P450 as well as phase II, phase III, and transporter enzymes could help to better evaluate the relevance of the in vitro models to detect hepatotoxic metabolites.

### 6.4. Refinement of DMPK Tools to Better Predict Clinical Outcomes

The dramatic improvements in clinical attrition rates due to poor pharmacokinetics (PK)/absorption, distribution, metabolism, and excretion (ADME) properties of small-molecule compounds that have been documented in the last two to three decades are an excellent opportunity to reflect on what may make initiatives successful [152]. These improvements were the combined results of significant investments in the field of ADME profiling and formulation, the more consistent inclusion of PK measurements in animal studies, the earlier integration of relevant ADME endpoints in discovery testing funnels (now routinely conducted in parallel to potency measurements), the development of targeted in silico filters, and some early initiatives to reach a consensus among scientists in academia and industry around optimization of ADME properties. High-throughput ADME profiling is now widely adopted within drug discovery R&D organizations or even provided by specialized contract research organizations [153]. Over the years, these ADME profiling platforms have been refined in terms of quality of assays and timing of assay execution, as well as by regular addition to the testing battery of additional assays with proven utility.

While the ADME profiling experience is worth mentioning and learning from, it should however be pointed out that significant differences exist with DILI prediction and these differences highlight the complexity behind DILI prediction, in particular iDILI. Firstly, in silico or in vitro ADME prediction can rapidly be validated in relevant animal models at reasonable cost and sufficient throughout in contrast to most DILI cases. Likewise, interrogation of PK in the clinic is rapid and simple, such that compound PK characterization and selection can occasionally take place in the clinic (one of the arguments for the use of exploratory INDs). This rapid feedback allows for the generation of in vitro-in vivo (IV-IV) correlations that markedly strengthen the validity of and confidence in in silico or in vitro predictions. Secondly, the basic mechanisms and principles behind ADME mechanisms are relatively well understood and this contrasts with the complexity, lack of full characterization or understanding, and diversity of mechanisms of DILI. A better alignment around the fundamentals of a biological phenomenon should clearly facilitate consensus reaching in a scientific field, as well as the definition of what endpoint or property is relevant to interrogate for prediction. Regular interactions and argumentations around mechanisms of DILI at scientific venues illustrate the state of our current knowledge of DILI: it is clearly difficult to efficiently predict a phenomenon that one does not comprehend totally.

Keeping these limitations in mind, it is noteworthy that some assays or models designed to predict DILI could be much better understood in terms of performance if precompetitive evaluation and standardization of experimental conditions and dosing paradigms would occur. This is one of the aspirational objectives of some recent initiatives such as MIP-DILI (see Section 4 for more details). The positive outcome would not be limited to the better conduct and interpretation of early high-throughput assays that could be conducted in parallel to ADME profiling; it could also demonstrate the lack of utility of tests currently used by some R&D organizations or lead to a better positioning of tests within a discovery testing cascade. For example, there is still quite a lot of debate around the utility and timing of tests for reactive metabolite formation or effects on mitochondrial function. Finally, evaluating new technologies is time consuming and often libraries of test articles in individual companies are too limited in size to generate meaningful testing and validation sets. Development, evaluation, and interrogation of these novel technologies would be much more efficient in the context of precompetitive efforts.

## 7. Concluding Remarks

Since DILI is a major cause of attrition during early and late-stage drug development, there is a need to develop reliable in silico, in vitro, and in vivo assays for better predicting hepatotoxicity in both animals and humans early in drug development. The present paper identifies some of the key opportunities and challenges that the pharmaceutical industry is facing with a focus on the in vitro DILI field. Scientists from academia and industry need to work closely together to standardize the use of the most promising tools, taking into account some of the practical considerations highlighted in this paper. Successful initiatives in other domains and, in particular, ADME, genetic toxicology, and microarray, should be used to guide future efforts and help to harmonize current and emerging models as well as strategies such as integrated risk assessment and mitigation plans at early stages of drug development. The current evolution in in vitro technologies stemming from decades of previous experience is opening an optimistic window on the future and authorizes hope for the alleviation of many of the limitations described in this review. After all, “*the future depends on what you do today,*” Mahatma Gandhi.

## Figures and Tables

**Table 1:** Examples of in vitro assays used in DILI prediction.

Cell model	Endpoints assessed	References
HepG2 cells	High content screening of cell viability	[61, 70, 119]
HepG2 cells	Mitochondrial injury	[78, 154]
Human liver-derived cell lines expressing human P450s	Cell viability	[63, 155–157]
Isolated primary human hepatocytes	High content screening of cell viability	[60, 158, 159]
Isolated primary rat hepatocytes	High content screening of cell viability	[49]
Isolated rat or human primary hepatocytes	Biliary efflux inhibition	[160–162]
HepaRG cells	High content screening of cell viability, BC dysfunction, intrahepatic cholestasis, cell viability, steatosis	[106, 163–165]
Membrane vesicle expressing bile salt export pump (BSEP)	BSEP activity inhibition	[19, 166, 167]
Isolated human primary hepatocytes	Covalent binding of radiolabeled compounds to proteins	[48, 168, 169]
Human hepatocytes plus cytokines	Cell viability	[130]
Hepatocytes (various species cocultured with nonparenchymal hepatic cells)	Liver cell viability and function	[29, 62]
Micropatterned human or rat hepatocyte/accessory cell cocultures	Cell viability function	[71]
Human liver microtissues	Cell viability	[30]
Human liver cell 3D microfluidic liver model	Cell toxicity (multiparametric)	[32]
